# Infrared camouflage in leaf-sitting frogs: a cautionary tale on adaptive convergence

**DOI:** 10.1098/rsif.2024.0771

**Published:** 2025-04-09

**Authors:** Devi Stuart-Fox, Katrina Joanne Rankin, Madeleine Shah Scott, Lu-Yi Wang, Amanda M. Franklin

**Affiliations:** ^1^ School of BioSciences, The University of Melbourne, Parkville, Victoria, Australia; ^2^ The University of Manchester, Manchester, UK

**Keywords:** infrared camouflage, thermoregulation, near-infrared, heating rates, background matching, convergent evolution

## Abstract

Many cryptic green animals match leaves in invisible near-infrared (NIR) wavelengths. This observation is an enduring puzzle because animals do not see NIR light, so NIR background matching is unlikely to contribute to visual camouflage. Two alternative explanations have been proposed—infrared camouflage (i.e. matching the temperature of the background) and thermoregulation—but neither hypothesis has been experimentally tested. To test these hypotheses, we developed bilayer coatings that mimicked the reflectivity of green leaf-sitting frogs with high NIR (HNIR) or low NIR (LNIR) reflectance. Under a solar simulator in the laboratory, agar model frogs with LNIR reflectance heated up more quickly and reached higher temperatures than those with HNIR reflectance. However, when placed in a tropical rainforest (natural habitat of leaf-sitting frogs), HNIR and LNIR models did not significantly differ in the similarity of surface temperature to the adjacent leaves or in core temperature, thus failing to support the infrared camouflage and thermoregulation hypotheses, respectively. The lack of difference between treatments is probably due to the limited exposure of frogs to direct solar radiation in their natural habitats. We propose an explanation for NIR background matching based on specific mechanisms underlying green coloration and translucence in frogs and caution against assuming adaptive convergence.

## 1. Introduction

Many animals are green and appear camouflaged against a background of green leaves. A universal feature of green leaves is that they reflect a high proportion of near-infrared (NIR; 700−2500 nm) light due to the light scattering properties of leaf cellular structures [1–3]. Curiously, some green animals, such as some leaf-sitting frogs, match the reflectance of leaves in NIR as well as visible wavelengths [4–6], even though no animal that we know of can see NIR light [7–10]. This NIR background matching remains an unsolved puzzle.

Two hypotheses to explain NIR reflectance matching are infrared camouflage and thermoregulation [5]. An animal’s reflectance properties influence the absorption of ultraviolet-visible (UV-Vis) (300−700 nm) and NIR (700−2500 nm) incident solar radiation, thereby affecting heat gain [11–13]. Matching reflectance properties of the background could improve the temperature match between an animal and the background. This may provide infrared camouflage from predators that hunt by sensing heat (i.e. greater than approx. 8000 nm), such as some snakes [14,15] and insects [16,17]. A non-mutually exclusive hypothesis is that animals at risk of overheating may have high NIR (HNIR) reflectance to aid thermo- and hydro-regulation [11,18–20], coincidentally matching the HNIR reflectance of leaves. Both these hypotheses require ecological settings where the animal is exposed to enough direct or indirect solar radiation to significantly influence heat gain. However, experimental tests of these hypotheses are lacking due to the difficulty of manipulating NIR reflectance independently of visible colour.

HNIR reflectance in leaf-sitting frogs was first documented in the Australian tree frog, *Litoria caerulea*, by Hugh Cott in his seminal 1940 book on ‘Adaptive coloration in animals’ [21]. Subsequently, Schwalm *et al*. [4] published a paper in *Science* reporting HNIR reflectance in species of neotropical leaf-sitting frogs belonging to two different families (glass frogs in the family Centrolenidae, and phyllomedusine tree frogs in the family Hylidae, subfamily Phyllomedusinae) and low NIR (LNIR) reflectance in other North American frogs, including some Hylidae species. The match between leaf NIR reflectance and frogs with HNIR reflectance has since been extended to more species [5,6,22], and its occurrence in multiple distantly related species has been used as evidence for convergent evolution of NIR matching and an adaptive function [5]. However, currently no empirical evidence exists for an adaptive function of NIR background matching in leaf-sitting frogs—or any other green animals.

Simulations based on a detailed thermoregulatory model of leaf-sitting frogs showed that under a scenario of 65% shade, HNIR and LNIR reflecting frogs differ by 0.5°C in maximum temperature reached and 0.4 h in maximum exposure time before needing to rehydrate. This difference was not enough to change the number of times the frog would need to rehydrate between dawn and dusk [6]. In addition, leaf-sitting frogs with HNIR reflectance are rarely exposed to direct sunlight because they are nocturnal and forage on wet or humid evenings [23,24]. During the day, they shelter in a wide range of shaded locations from the crowns of trees to low vegetation [23,25], which may expose them to reflected light from vegetation and occasional sunspots. Thus, it remains unclear whether the observed reflectance difference results in a biologically meaningful difference in the temperature of frogs under natural conditions.

To address this problem, we developed a technique to independently manipulate visible and NIR reflectance using a bilayer coating inspired by recently developed coatings for passive cooling [26,27]. Using this technique, we created coatings that mimicked the reflectivity of green NIR background matching (HNIR reflectance) and green NIR background contrasting (LNIR reflectance) leaf-sitting frogs (families Hylidae and Rhacophoridae; [5]). We applied the coatings to fabricated agar frogs and quantified temperature differences produced by our treatments (HNIR and LNIR reflectance) under controlled laboratory conditions. We then tested the infrared camouflage and thermoregulation hypotheses by deploying frog models in tropical rainforest, the natural habitat of many leaf-sitting frogs. The infrared camouflage hypothesis predicts that the surface temperature of HNIR (matched) frogs should better match the leaf substrate temperature compared with LNIR frogs, while the thermoregulation hypothesis predicts a significant difference in internal and/or surface temperature between HNIR and LNIR frogs.

## 2. Methods

### 2.1. Frog models: fabrication

**Figure 1.**
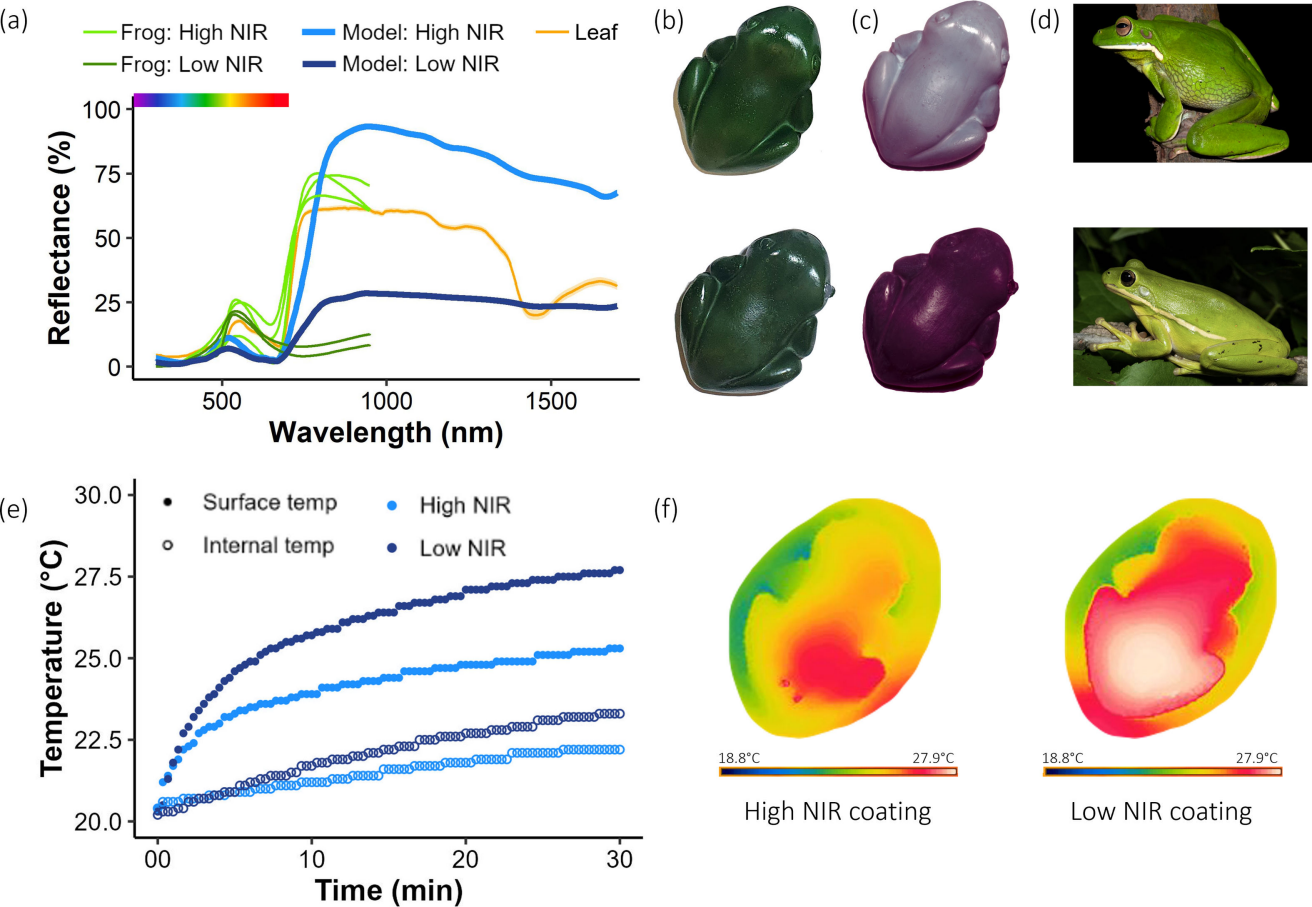
Validation of experimental treatments. (a) reflectance measurements of models (thick blue lines) resemble published spectra of leaf-sitting frogs (thin green lines [6]), with a peak in green wavelengths and HNIR (light lines) or LNIR (dark lines) reflectance. Average spectra of six *Eucalyptus* leaves (*E. ficifolia, E. regnans* and *E. saligna*) included for comparison; (b) photograph of models through a visible filter (400−700 nm; top: HNIR reflectance, bottom: LNIR reflectance); (c) photograph of models through NIR filter (700−1000 nm; top: HNIR reflectance, bottom: LNIR reflectance); (d) examples of real frogs with HNIR (top; *Litoria infrafrenata,* image credit Jodi Rowley) and LNIR (bottom; *Hyla cinerea*, image credit Saunders Drukker) reflectance [5,6]. (e) internal and surface temperature heating rates of one small model placed in thermal chamber; (f) thermal image of models (FLIR T420; left: HNIR, right: LNIR reflectance; under a halogen light source).

We made agar frog models of two sizes with coatings that had similar green reflectance but either HNIR or LNIR reflectance (figure 1). The frog models were cast in agar gel (Mycelium Emporium) of ratio 20 g agar powder : 500 ml water to ensure they would retain their form when subjected to a range of temperature and humidity levels. To independently control visible and NIR reflectance, we drew inspiration from bilayer coatings, which comprise a lower layer that efficiently scatters light including NIR wavelengths, and an upper layer that filters certain wavelengths to produce the desired colour [26,28]. Model frogs were first sprayed with a commercial primer (Tough Stain Blocker; Taubmans) to prevent the agar sweating and interacting with the coatings. We then hand-painted models with Solacoat in ‘off white’ as the lower layer for the HNIR coating and ‘neutral grey’ acrylic paint (Jasart) for the LNIR coating. In both cases, the upper layer comprised an airbrushed mix of clear brown and clear green acrylic paints (PL24 and PL20, respectively; SMS Paints). Coatings reduce evaporative cooling; therefore, we included a control group of frog models which were uncoated, but the agar tinted green with food dye (Queen Rainbow Food Colours, Australia). Thus, in total, we had three treatment groups: HNIR, LNIR and uncoated (U). For each treatment group, we made frog models of two sizes to resemble leaf-sitting frogs found in the tropical rainforests of our field site in northeastern Australia; dainty green tree frog (*Litoria gracilenta*; 45 mm snout–vent length (SVL); [24]) and the white-lipped tree frog (*Litoria infrafrenata*; 110 mm SVL; [24]).

To visualize the differences in the reflectance properties of the treatments, we photographed the three treatments (HNIR, LNIR and U) using a full-spectrum fused-silica converted Nikon D7200 dSLR camera (Camera Clinic, Melbourne) affixed with a 60 mm UV-Vis-IR lens (CoastalOpt, JenOptik, USA). Photographs were taken using combinations of optical filters (Edmund Optics, Singapore): visible (400−700 nm; filter item numbers 84 754 and 84 727) and NIR (700−1100 nm; filter item number 84 760 and 84 735; figure 1). We used a flash unit (Nikon SB140 UVIR clone; Beyond Visible, USA) for visible photographs and a 500 W halogen lamp (Arlec, Australia) for NIR.

### 2.2. Frog models: validation

Heating rate is proportional to the amount of energy absorbed by a surface. This is related to both the reflectance properties of the surface, as well as the irradiance spectrum of the light source [11,29]. Therefore, we used reflectance measurements of the model frogs or real frogs and the irradiance spectrum of sunlight (ASTM G-173; [30,31]) to calculate the summed energy flux, also known as reflectivity, *R* [12]. Specifically, reflectivity is the ratio of incident light to reflected light, integrated over the wavelength range of solar radiation (i.e. 300–2600 nm),


(2.1) $$R=\;\frac{\int_{i}^{n}S\left(\lambda \right)I\left(\lambda \right)d\lambda }{\int_{i}^{n}I\left(\lambda \right)d\lambda },$$

where S is the reflectance of the model frog or real frog and I is the solar irradiance from wavelength (λ) i to n.

To quantify that the difference in reflectance between our treatments was similar to real leaf-sitting frogs, we measured the reflectance of models using a spectrometer (figure 1) and compared them with published reflectance spectra of leaf-sitting frogs from Herrerías-Azcué *et al*. [6]. The reflectance of model frogs was recorded from 300 to 1700 nm (98.9% of solar energy; figure 1) using a Flame UV/Vis spectrometer (300−1000 nm; Ocean Optics Inc.) and a NIRQuest spectrometer (1000−1700 nm; Ocean Optics Inc.). To provide illumination across the entire spectrum we used both a PX2 pulsed xenon light source (Ocean Optics Inc.) and a HL-2000 tungsten-halogen light source (Ocean Optics Inc.). The spectrometers and light sources were connected with a quadrifurcated fibreoptic, and reflectance was measured with the probe mounted in a probe holder (RHP1, Ocean Optics) with illumination and reflection at 45° to the model surface. Measurements were calibrated to a Spectralon 99% white reflectance standard (LabSphere, NH, USA). To investigate whether specular reflectance may contribute substantially to overall reflectance, we also measured hemispherical reflectance (i.e. reflectance integrated over 180°; [12]) using an integrating sphere with an inbuilt tungsten-halogen light source (ISP-REF; Ocean Optics Inc., Dunedin, FL, USA). The integrating sphere was connected through a bifurcated fibreoptic to the same spectrometers as above. As average reflectance spectra were the same using both approaches, we used the former because it included UV wavelengths (the inbuilt tungsten-halogen light source of the integrating sphere does not emit UV, so the measurement wavelength range was limited to 400–1700 nm). We report measurements of HNIR and LNIR frog models compared with real frogs (see §3.1). Uncoated agar models were transparent with uniformly low reflectance.

Reflectance data for five real frogs (two with LNIR reflectance and three with HNIR reflectance) were extracted from Herrerías-Azcué *et al.* [6] (figure 1). These data extended from 300 to 950 nm, which accounts for 75.4% of solar energy. NIR wavelengths from 950 to 1700 nm account for a significant proportion of solar energy (23.5%). Therefore, we extended the reflectance measurements to 1700 nm with two different approaches. The first fitted reflectance as an exponential decline from the value at 950 nm, following the approach by Herrerías-Azcué *et al*. [6] to obtain reflectivity estimates used in their biophysical models (electronic supplementary material, figure S1). The second approach fitted a linear extension by continuing the slope from 850 to 950 nm (electronic supplementary material, figure S1). This produced negative values for one spectrum, which were changed to zeros. We then calculated and compared these three reflectivity values between model and real frogs (table 1): reflectivity from actual data (300–950 nm; 75.4% solar energy), reflectivity with exponential decline (300−1700 nm; 98.9% solar energy), or reflectivity with linear extension (300−1700 nm; 98.9% solar energy).

**Table 1.** Reflectivity of real frogs and model frogs and differences between high and low NIR specimens. *Notes*. Real frog reflectance data were limited to 300–950 nm [6]. We calculated reflectivity for this range, and we also extended the frog reflectance curves to 1700 nm with either exponential decline (following [6] or linear extension (electronic supplementary material, figure S1). Reflectivity values for model frogs from 300 to 1700 nm are measured rather than extrapolated values.

frog species or model type	reflectivity_300–950 nm_	reflectivity_300–1700 nm_ exponential decline	reflectivity_300–1700 nm_ linear extension
high NIR model	24.7	39.5	39.5
*Cruziohyla calcarifer*	26.0	26.1	27.7
*Cruziohyla craspedopus*	33.3	32.6	39.7
*Phyllomedusa sauvagii*	29.2	28.4	33.3
low NIR model	9.5	13.7	13.7
*Gastrotheca riobambae*	10.7	9.4	13.2
*Hyla cinerea*	7.8	6.8	9.7
average difference (high–low)			
models	15.2	25.8	25.8
frogs	20.2	20.9	22.1

#### 2.2.1. Laboratory heating rates experiment 1

To confirm that our model coatings differed in their radiative heating properties, we measured the model heating rate following methods described in [11,12]. Briefly, small models of HNIR and LNIR were placed in a closed glass thermal chamber surrounded by flowing water (18°C) to control the ambient temperature within the chamber. The circulating water is controlled for conduction and convection when exposed to radiation from a solar simulator light source. We illuminated the chamber from directly above using a solar simulator light source (Sciencetech UHE-NS) with an energy intensity of 1000 W m^−2^. This simulates the power and irradiance spectrum of the sun ranging from 250 to 2500 nm (AM1.5G). Although solar intensity varies latitudinally and throughout the day, this indicative intensity allowed us to confirm whether our treatments can affect radiative heat gain. A window in the top of the chamber allowed us to insert different optical filters to control the illumination wavelengths in the chamber (full spectrum, 300–1700 nm; UV-Vis only, 300–700 nm; or NIR only, 700–1700 nm). Inside the chamber, samples were placed in the centre on a transparent acrylic platform. One thermocouple was placed on the surface and another inserted into the middle of the agar frog to record core temperature, while a third thermocouple in the chamber recorded air temperature. The thermocouples were connected to a thermometer and the temperatures were recorded once every 20 s. Each model was subject to the same heating and cooling regime: 5 min cooling, 30 min full-spectrum heating (UV-Vis and NIR), 15 min cooling, 30 min NIR only heating, 15 min cooling, 30 min UV-Vis only heating, 15 min cooling. The initial 5 min cooling period without any illumination was to ensure that the sample temperature was stable prior to the first heating period; the 15 min cooling period between heating was to ensure samples were of similar temperatures at the beginning of the heating periods.

#### 2.2.2. Laboratory heating rates experiment 2

Due to size constraints of the chamber, we were only able to test the small-sized models following the protocol described above. To compare large and small models, we measured heating rates of both large and small models for HNIR, LNIR and uncoated directly under the solar simulator with no optical filters (i.e. full spectrum illumination). These measurements do not control for effects of convection and conduction, but together with the chamber experiments, provide an indication of relative differences in radiative heating between treatments. We recorded surface temperature and core temperature when exposed to full spectrum (300–1700 nm), NIR only or UV-Vis only radiation for 30 min. From the heating rate data, we extracted the maximum temperature reached at the end of the heating period and calculated heating rate as the slope coefficient from a linear model fitted to the first 2 min of heating (table 2).

**Table 2.** Heating rate and maximum temperature for model frogs measured in laboratory experiments. *Notes*. Heating rate is calculated from the first 2 min of exposure to a light source. Experiment 1 (small models only) controls for conduction and convection, and measures heating rate under different lighting conditions: full spectrum (300–1700 nm), UV-Vis only (300–700 nm) and NIR only (700–1700 nm). Experiment 2 does not control for conduction and convection, but allows comparison between large and small models.

	high NIR		low NIR		uncoated	
	internal	surface	internal	surface	internal	surface
*experiment 1: chamber, small models only*						
*full spectrum*						
maximum (°C)	22.2	25.3	23.3	27.7		
heating rate (°C min^–1^)	0.118	0.879	0.161	1.404		
*NIR only*						
maximum (°C)	21.1	22.2	21.8	24.2		
heating rate (°C min^–1^)	0.000	0.257	0.000	0.600		
*UV-Vis only*						
maximum (°C)	20.6	21.9	20.6	22		
heating rate (°C min^–1^)	0.000	0.311	0.000	0.332		
*experiment 2: no chamber*						
*small models*						
maximum (°C)	22.8	26.4	23.8	27.8	21.9	23.3
heating rate (°C min^–1^)	0.129	0.846	0.161	1.104	0.064	0.525
*large models*						
maximum (°C)	22.6	26.1	22.6	27.3	21.8	23.8
heating rate (°C min^–1^)	0.064	0.750	0.086	1.061	0.032	0.525

### 2.3. Field experiments

We conducted our field experiment at the Daintree Rainforest Observatory (16°6′14′′ S, 145°26′58′′ E) for three days in October 2022. This month is during the peak spring–summer activity period of the frogs (September–February; [23]) and represents a period when selection related to the reflectance of solar radiation is likely to be relevant. Based on average monthly climate data from Worldclim [32], October has the highest average incident solar radiation, low precipitation and vapour pressure and moderate-to-high average temperature (see electronic supplementary material, figure S2 for monthly climate variables for the site).

Each day, we selected five sites, and at each site, we placed model frogs in pairs on a leaf. Our primary goal was to compare HNIR and LNIR treatments under the same conditions (same background and microclimate); therefore, we paired HNIR and LNIR treatments on the same leaf (large LNIR with large HNIR; small LNIR with small HNIR). The small and large uncoated frogs were paired together to compare the effect of evaporative cooling on differently sized frogs. We chose sites that were likely to resemble the microhabitat of shaded shelter sites used by leaf-sitting frogs during the day [23,25], which is when incident solar radiation has the potential to influence heat gain. Both *L. gracilenta* and *L. infrafrenata* are arboreal and occur in a wide range of wet forest vegetation types and heights (including domestic gardens in the case of *L. infrafrenata*). Our sites were necessarily in the lower strata of the forest (heights that we could reach approx. 1–1.5 m above ground), in shaded position but with some dappled light such that occasional sunspots could potentially illuminate our models.

Model frogs were deployed in the morning (9.00–11.00) and collected each afternoon (16.00–17.00) to minimize damage by desiccation and nocturnal animals. The internal temperature of each model frog was recorded every 5 min using a thermochron iButton (DS1921G, Analog Devices Inc., MA, USA) embedded into the agar, and air temperature was measured with an iButton placed near the models. To measure the surface temperature of the model frogs and the leaf substrate, we used a FLIR thermal camera (T420, Teledyne FLIR, USA) and took a thermal image of each pair of frogs twice a day (11.00–12.00 and 15.00–16.00; electronic supplementary material, figure S3). We extracted the average surface temperature of each model and the adjacent leaf using FLIR Thermal Studio (version 1.9.40.0).

### 2.4. Statistical analysis of field experiments

To assess whether frog model surface temperature matched adjacent leaf surface temperature (infrared camouflage hypothesis) we used a linear mixed-effects model (LMM; lmer function from lme4 package; [33]). The response variable was the difference between frog surface temperature and adjacent leaf temperature. NIR coating (high, low or uncoated), size (small, large) and the interaction were included as fixed effects. All frogs were paired in the field and there were multiple pairs at each site; therefore, we included pair ID and site ID as random effects.

To investigate the effect of NIR coating and model size on maximum internal temperature, we extracted daily 95th percentiles for each model from the iButton recordings. We used the 95th percentile to avoid outliers that could influence results but may not reflect actual treatment differences (e.g. short exposure to a sunspot for one model but not the adjacent one), although results were qualitatively the same using maximum values. We ran an LMM with NIR coating, size and the interaction between these variables as fixed effects, and included pair ID, site ID and iButton ID as random effects.

It is possible that within pairs of frogs one type of model frog is consistently warmer than the other type. To assess this, we linked the time series iButton temperature data for pairs of frogs, and then calculated the difference between LNIR and HNIR reflectance model frogs (coated). We ran an LMM (lme function from nlme package; [34]) with temperature difference as the response variable, air temperature and size as predictor variables, site ID and pair ID as random effects, and an AR1 autocorrelation structure. For the uncoated frogs, we calculated the difference between the large and small frog and ran the same model with temperature difference as the response variable but without size as a predictor. If there are consistent differences between frog model types, we would expect the maximum likelihood estimate (MLE) of the intercept to be significantly different from zero.

For all models, the significance of fixed effects was determined using Wald’s chi-square tests, and differences among groups were assessed using MLEs, effect sizes and 95% CIs [35,36]. Model fit was assessed using diagnostic plots. All analyses were conducted using R v. 4.3.0 [37].

## 3. Results

### 3.1. Frog models: validation

The reflectance of our HNIR and LNIR treatments was similar in visible wavelengths but differed markedly in NIR. Although the spectral profiles of our painted frog models differed from those of real frogs (figure 1, electronic supplementary material, figure S1), total energy flux (i.e. reflectivity), which is the parameter relevant to our hypotheses, was very similar (table 1). Based on published spectra (wavelength 300–950 nm), the reflectivity of frog species with HNIR reflectance ranges from 26.0 to 33.3% and species with LNIR reflectance ranges from 7.8 to 10.7%. The reflectivity of the HNIR frog models was 24.7% and the LNIR models was 9.5% over this wavelength range. If we consider the wavelength range, we measured for the models (300–1700 nm), which accounts for 98.9% of solar energy, and assume either exponential decline or linear extension of reflectance spectra of real frogs, reflectivities of our models are even closer to those of real frogs (table 1). The method used to extend the reflectance spectra of real frogs had minimal impact on the difference in reflectivity between HNIR and LNIR frogs (less than 2%). These values indicated that our models were similar in reflectivity to real leaf-sitting frogs based on available data (table 1), and can therefore approximate radiative heat gain of similar-sized leaf-sitting frog

In experiments conducted within a chamber controlling for convection and conduction, LNIR reflectance model frogs heated up more quickly and reached higher temperatures than HNIR reflectance model frogs (figure 1, table 2). This was documented for both internal and surface temperature, although there was a greater difference between treatments in surface temperature (figure 1, table 2). We confirmed that the observed temperature difference was due to the difference in NIR reflectance because effects were observed under NIR or full spectrum illumination, but not under UV-Vis illumination (table 2). Results were similar for small and large frogs (experiments with large frogs did not control for convection and conduction) except that internal temperature did not differ between HNIR and LNIR treatments for large model frogs (table 2). Uncoated frogs heated up more slowly and reached notably lower surface and internal temperatures than coated frogs due to the effect of evaporative cooling (table 2).

### 3.2. Field experiments

**Figure 2.**
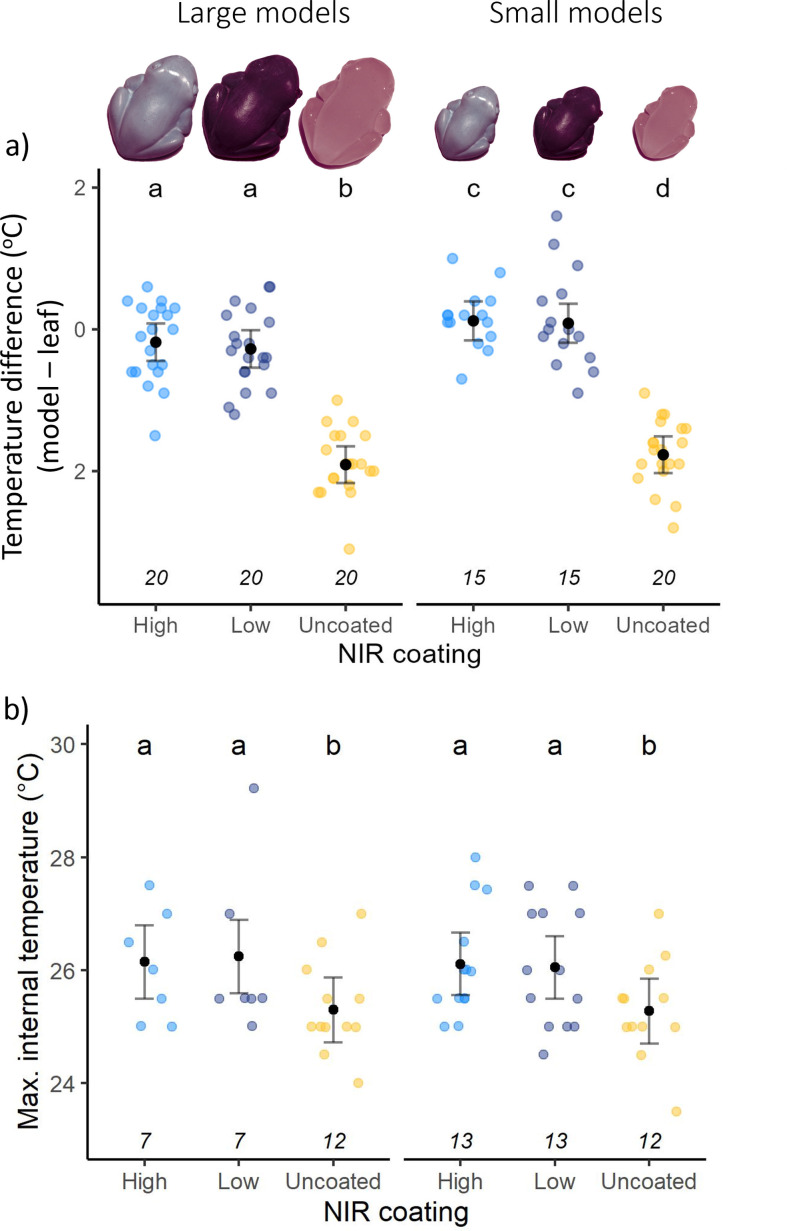
Surface and internal temperature of large (left) and small (right) model frogs that were either uncoated, or coated with LNIR or HNIR reflective paint. (a) Infrared camouflage hypothesis. Difference between model surface temperature and adjacent leaf temperature extracted from thermal images collected at the field site. Overall, large models were cooler than small models, regardless of treatment, and the uncoated models (large and small) were cooler than the adjacent leaf temperature. The coated models (LNIR and HNIR) did not differ significantly from the adjacent leaf temperature. (b) Thermoregulation hypothesis. Maximum internal temperature of model frogs, calculated as the 95th quantile from the iButton data for each model frog for each day. The maximum internal temperature of uncoated models was lower than coated models (LNIR and HNIR) and there was no impact of size. Black points indicate MLEs, error bars show 95% CIs and coloured points indicate raw data. Letters indicate statistically different groups, sample sizes indicated in italics above the coating type.

Surface temperatures of model frogs differed significantly between treatments (χ^2^ = 150.40, d.f. = 2, *p* < 0.001). Uncoated model frogs were significantly cooler than the adjacent leaf, but LNIR and HNIR treatments were not significantly different and were similar to the temperature of the adjacent leaf (figure 2a; electronic supplementary material, figure S3). Large model frogs had cooler surface temperature than small frogs (χ^2^ = 4.24, d.f. = 1, *p* = 0.039), but there was no difference between large and small frogs in the effect of the NIR coating type (interaction term: χ^2^ = 0.94, d.f. = 2, *p* = 0.62).

Similar to the surface temperature of the model frogs, there was no difference in internal temperature between LNIR and HNIR reflectance model frogs and uncoated model frogs were significantly cooler than coated models (χ^2^ = 22.07, d.f. = 2, *p* < 0.001; figure 2b). There was no effect of model size on maximum internal temperature (χ^2^ = 0.18, d.f. = 1, *p* = 0.67) and no interaction between size and coating type (χ^2^ = 0.26, d.f. = 2, *p* = 0.88). We verified these results by comparing the difference in temperature between paired frogs, which had the same background and microclimate conditions. We detected no consistent difference in temperature (i.e. CIs cross-zero) between model frog pairs (small LNIR versus small HNIR; large LNIR versus large HNIR; small uncoated versus large uncoated) and this was the case across all environmental temperatures (electronic supplementary material, figure S4).

We identified two instances where both HNIR and LNIR models are likely to have been exposed to sunspots at the same time (electronic supplementary material, figure S5). These showed sharp temperature spikes in the iButton data for both models. Consistent with laboratory data, in these instances, the LNIR models reached approximately 1–1.5°C higher temperature than the HNIR models over a period of 15–20 min.

## 4. Discussion

The adaptive significance of NIR background matching is an enduring puzzle. Here, we independently manipulated animal-visible and NIR reflectance for the first time to test adaptive hypotheses for this phenomenon. We created model frogs that closely resemble the measured reflectance of leaf-sitting frogs that do and do not exhibit NIR background matching. The difference in internal temperature of our coated models due to radiative heating (controlling for conduction and convection) was 1°C under a solar simulator at 66% sun intensity, which corresponds well to the maximum temperature difference of 1.7°C estimated from biophysical models for more extreme reflectance differences under full sun [6]. However, when these models were placed in natural rainforest conditions, we detected no significant difference between HNIR and LNIR model frogs in internal or surface temperature. Although our model frogs closely approximate the biophysical properties of real leaf-sitting frogs, we found no support for either the thermoregulation or infrared camouflage hypotheses.

The thermoregulation hypothesis predicts that the difference between HNIR and LNIR reflectance should result in NIR models having higher body temperatures. This assumes that the frogs are exposed to sufficient direct or indirect NIR radiation to affect body temperature. As leaf-sitting frogs prefer shaded microhabitats [6,38–40], we placed our models in realistic conditions in shade or dappled light. It is possible that frogs experience brief exposures to sun (e.g. sunspots in forest, which quickly move), but our results suggest that brief exposures, together with indirect radiation, are not sufficient to affect core body temperature. This may be particularly true for larger species due to higher thermal inertia, which is supported by our laboratory experiment with large models that found no difference in core temperature after 15 min exposure. Our results suggest that differences in NIR reflectance are unlikely to influence thermoregulation of leaf-sitting frogs due to a lack of exposure to sufficient direct and indirect radiation [4,6,38,41].

Although NIR reflectance may have little effect on body temperature most of the time, it may confer a benefit in specific circumstances and consequently be under selection. Specifically, the diurnal shelter locations of frogs may occasionally be exposed to sunspots, which could cause the frogs to experience thermal stress for brief periods. In our temperature logger dataset, we identified two likely instances in which both HNIR and LNIR models were exposed to sunspots simultaneously. Although LNIR models reached a higher temperature in these two instances (LNIR versus HNIR: instance no. 1: 29 versus 27.5°C; instance no. 2: 33 versus 32°C; electronic supplementary material, figure S5), the maximum temperature reached and duration (approx. 10 min greater than or equal to 1°C difference) are unlikely to cause thermal stress [42–44]. Additionally, we placed models on leaves; whereas daytime shelters chosen by frogs, which are most active at night, are likely to be less exposed to direct or indirect sunlight. In contrast to our models, a live frog can also move out of a sunspot, although moving from its shelter may attract the attention of predators. Therefore, based on our data and the ecology of leaf-sitting frogs, it seems unlikely that NIR reflectance is under strong selection for thermoregulation.

The infrared camouflage hypothesis predicts that a closer match in reflectance to the background should correspond to a closer match in temperature, affording protection from heat-sensing predators. Our results show no difference between HNIR and LNIR treatments in temperature matching to the background. Tropical rainforests are home to snakes with heat-sensing organs (Pythonidae, Viperidae; [14,45]), but these may not impose a strong selective pressure on frog NIR reflectance for thermal matching. Most species of python and many vipers that inhabit rainforests are primarily active at night [46–50], so heat gain from absorption of NIR light would be minimal during the activity period of these predators. Additionally, the effect of evaporative cooling for our models was substantial, with uncoated models roughly 2°C cooler than the adjacent leaf. The coatings on our models would have allowed minimal evaporation, and would have maximized any detectable differences in radiative heating. Frogs vary greatly in evaporative water loss through the skin, depending on the environments in which they live. While species such as glass frogs may have high evaporative water loss, many other species have waxy coatings that minimize water loss [51,52]. For frog species with high evaporative water loss it is likely that evaporative cooling would override any thermal effects of reflectance and create a thermal mismatch to the background, as observed in our uncoated models.

If NIR matching is not due to selection for thermoregulation or infrared camouflage, why do some frogs have HNIR reflectance? It may be related to the mechanism used by frogs to produce a green appearance, with NIR matching or contrast to leaves being a coincidental consequence. In frogs, skin colour is produced by a combination of different types of pigment cells (chromatophores) in the skin. Green arises from absorption by pigments in xanthophores, combined with light scattering by iridophores and/or connective tissue [4,5,41,53,54]. For example, in *Hyla cinerea*, a green frog with LNIR reflectance, green is produced by yellow pigments in xanthophores, combined with ordered crystals that reflect blue light in iridophores [55]. Melanophores beneath the xanthophores and iridophores contain melanin pigment, which absorbs remaining wavelengths, including NIR. By contrast, the melanophores of some leaf-sitting frogs contain a unique wine-red pteridine pigment, pterorhodin [56]. Unlike melanin, pterorhodin does not absorb NIR wavelengths. The presence of pterorhodin instead of melanin results in HNIR reflectance because the non-absorbed NIR light is scattered by underlying connective tissue, which produces broadband scattering. Pterorhodin has been found in all Australo-Papuan hylid frogs (subfamily Pelodryadinae) examined to date (41 species; [56]), as well as all New World phyllomedusine hylids (subfamily Phyllomedusinae). Phylogenetically, these two subfamilies are sister lineages with a common Gondwanan ancestor and are distinct from all other subfamilies within the Hylidae [57,58]. Thus, it is probable that the use of pterorhodin for skin pigmentation evolved only once in hylid frogs, rather than arising repeatedly through convergent evolution.

The other family of frogs with HNIR reflectance, the glass frogs (Centrolenidae), present a different mechanism. These frogs are among numerous green frog species with translucent skins, in which melanophores and other chromatophore cells are completely or partially absent. In the absence of dermal chromatophores (i.e. in the absence of pigment cells), these species have convergently evolved an alternative mechanism to produce green. In translucent species, green coloration arises from proteins in the lymph and interstitial fluid that bind the blue-green pigment, biliverdin, and modulate its spectral absorbance [54]. Biliverdin-binding serpins (BBSs) do not absorb in the NIR, and the non-absorbed NIR light is scattered by connective tissue. BBSs have evolved multiple independent times in frogs, likely to enable translucence [54]. Thus, the occurrence of NIR background matching in frogs may not be the result of adaptive convergence as previously suggested [5]. Instead, we suggest that NIR background matching is a coincidental consequence of the evolution of pterorhodin in the common ancestor of Pelodryadinae and Phyllomedusinae, and convergent evolution of translucence in other frog families.

Although NIR reflectance may not have adaptive significance for leaf-sitting frogs, it may nonetheless be important in many other species, including frogs, that are more frequently exposed to direct sunlight. For example, some frog species (e.g. reed frogs) aestivate in hot arid environments and can be fully exposed to the sun during this time [59], while other frogs are known to bask in full sun, changing colour to modulate body temperature [60]. The adaptive significance of NIR reflectance has not been examined in these species, but there is increasing experimental and comparative evidence that NIR reflectance is important for thermoregulation in lizards [61], butterflies [18,19], beetles [11], intertidal gastropods [13] and birds [20]. Equally, however, convergence in NIR reflectance in some island songbirds appears to be non-adaptive [62]. Our study cautions against assuming adaptive convergence and proposes a non-adaptive solution—that it is a coincidental consequence of colour mechanisms—to the enduring puzzle of NIR background matching in leaf-sitting frogs.

## Data Availability

Data and analysis code are available on Dryad [63]. Supplementary material is available online [64].
